# Mr. Veitch, on Fracture of the Skull

**Published:** 1800-12

**Authors:** James Veitch

**Affiliations:** No. 20, Edgecumbe Street, Stonehouse


### *To the Editors of the Medical and Phyfical Journal.*

GENTLEMEN,

I Enclofe you a cafe, probably fomewhat remarkable for the rapidity of recovery, when the extent of injury and nature of climate in which it happened, the Weft Indies, fuppofed fo very unpropitious to the healing of wounds, are confidered.

The fuccefs attending this operation, as well as fome others equally important, may be in a great degree afcribed to our having many opportunities of procuring fupplies of frefh vegetables, which had the power of obviating predifpofition to fcurvy amongft the fhip's company I belonged to; a tendency of the habit to be guarded againft by every poffible means, where action is expected or energy required. I am,

GENTLEMEN,

Your obedient fervant,

JAMES VEITCH.

*No. 20, Edgecumbe Street, Stonehouse,*

*October* 20, 1800.

P. S. In my letter to the Commiffioners for Sick and Wounded Seamen, you have made a fmall miftake, by fubfcribing me *Veiten* inftead of *Veitch*, which you will oblige by correcting.

---

May 28, 1797, John Spencer, aged about twenty, in handing the main-top gallant fail, fell from the yard, and fractured the right parietal bone. On being informed of the accident, I could not help thinking my interference, from the height being fo very confiderable, 116 feet, would amount to no more than what is expected by the phyfician or furgeon, when they adminifter a placebo, merely to gratify relatives, confcious that their patient is irretrievably loft; but the refult fhews how erroneous opinions, formed without exact inveftigation of the extent of injury, may prove. He was ordered to be carried under under the half deck, the place appointed for the reception of the fick, and fortunately it was not crowded, there being only one man in the fick birth, on whom I had operated for Empyema. The fcalp prefented a wound rather irregular, running in fome degree in the direction of what is called the fuperior pofterior procefs of the parietal bone to that of the anterior inferior, and it was fo large as to admit no doubt that confiderable injury was inflicted on the fkull, for I found by the moft fuperficial examination, extenfive fracture with depreffion, attended with fymptoms which indicated this; laborious refpiration, coma, intermitting and flow pulfe. The wound inflicted by the fall was rendered more extenfive, and then conical, my knife acting in fubfervience to the higheft refpect for the covering parts. The fcalp when raifed, brought to light an extenfive fracture of the parietal bone, with depreffion and infraction, following the direction of the external wound which had been inflicted by the fall, and only connected firmly at one point, (which depreffed bone reminded me of a peninfula) but feveral parts of the undepreffed parietal bone followed the principal depreffion, and in themfelves formed each a fource of compreffion; features in the face of the fracture, that induced me to form the refolution of removing the depreffed piece of bone entirely, provided, after an application or two of the trephine, this piece, with the compreffing points, could not be elevated. The branches of the temporal artery were allowed to bleed freely, notwithftanding he had loft blood from the arm to ten ounces, which practice, the injury done the brain and membranes will juftify. Being unable to accomplifh my object, by attempting elevation to the extent mentioned, the trephine was applied four times at thofe points noticed; and as they were by thefe applications removed from acting on the brain, and the depreffed piece likewife much difengaged, I judged it time to infulate by dividing the connection with the parietal bone. On examining the depreffed piece of bone, I found the internal lamina fractured conically, and projecting from the line of health, and in itfelf capable of acting as a compreffing caufe on the brain.* The fcalp was now returned as nearly as poffible to its natural fituation, and the wound covered with light and fimple dreffing; he was put into a cot, as admitting more freely the circulation of air round the body, to carry off fuperfluous heat, than a hammock, and an opiate was adminiftered. He was laid on his left fide. This accident happened in the morning; and it is almoft unneceffary to fay,

I faw

* Even a part of the internal lamina was detached at the point laft divided.

I ſaw him repeatedly during the day; at night an anodyne draught was given him, and nothing unpleaſant occurred until about twelve o'clock, when I was informed Spencer was in a dangerous way. On viſiting I found him much convulſed, with intervals of eaſe, which cauſed me to order forty drops of laudanum, and this was repeated within an hour, without producing any good effect. The wound was undreſſed and examined, and I had the ſatisfaction to diſcover the cauſe of convulſion in this caſe, in a ſmall fragment of bone lodged partially in the ſubſtance of the brain, and which had eſcaped my notice in dreſſing the wound. Were not theſe convulſions proof of returning ſenſibility? and from which appearance, on taking away the hoſtile cauſe, was there not reaſon to infer more favourably? The removal of this foreign body was ſoon ſucceeded by tranquillity, and the following day he was able to give a diſtinct anſwer, and name the objects preſented to him.

℞. Fol. Sennæ, ʒvj. Crem. tart. ʒj. Fruct. tamarind, ℥j. Aq. font. Bul. ℥viij. Macera. per hor. dein. cap. ℥ij. omni 2ª hor. don. purg.

It would prolong this caſe to give a detail of his daily treatment. He was kept low while there was any thing to be apprehended from ſymptomatic fever; opiates combined with ſudorifics were given at night, and the wound and bowels regularly attended to. In a month from the date of the accident, the wound was almoſt completely healed, and he had walked about the ſick birth eight days, and been from bed daily for twelve. In this caſe, the dura as well as pia mater were not only injured, but ſuch was the force of the fall, that part of the brain was forced through the interſtices of the fractured bone. The trepan recommended by very eminent authority for commencing this operation, is not a very admiſſible inſtrument in operating at ſea, where the motion of the ſhip renders it ſo difficult to act with ſteadineſs, even with the trephine.

In injuries of the brain, where we ſuppoſe every thing required, is done by operating, and that are followed by convulſions, this caſe may not only offer a ſtrong inducement to examine the wound, in order to diſcover the cauſe of theſe morbid actions, but it points out the propriety of not deſerting ſuch accidents as a forlorn hope; becauſe from meaſurement of the extent of injury the brain has ſuſtained, it will frequently give riſe to a mode of reaſoning that may deceive the ſurgeon himſelf, and lead him to ſuppoſe the miſchief done his patient is not to be relieved by the reſources of his art.

